# Women’s health and well-being in low-income formal and informal neighbourhoods on the eve of the armed conflict in Aleppo

**DOI:** 10.1007/s00038-018-1150-2

**Published:** 2018-07-28

**Authors:** Balsam Ahmad, Fouad M. Fouad, Shahaduz Zaman, Peter Phillimore

**Affiliations:** 1 Institute of Health and Society, Medical Faculty, Newcastle University, Baddiley-Clark Building, Richardson Road, Newcastle upon Tyne, NE2 4AX UK; 2 Department of Epidemiology and Population Health, Refugee Health Program, Global Health Institute, American University of Beirut, Beirut, Lebanon; 3 Global Health and Infection Department, Brighton and Sussex Medical School, University of Sussex, Room 2.17, Medical School Teaching Building, Falmer, Brighton, BN1 9PX UK; 4 Geography, Politics and Sociology, Newcastle University, Claremont Bridge Building, Newcastle upon Tyne, NE1 7RU UK

**Keywords:** Syria, Conflict, Qualitative, Formal neighbourhoods, Informal neighbourhoods, Well-being, Urban policy, Married women

## Abstract

**Objectives:**

To explore how married women living in low-income formal and informal neighbourhoods in Aleppo, Syria, perceived the effects of neighbourhood on their health and well-being, and the relevance of these findings to future urban rebuilding policies post-conflict.

**Methods:**

Semi-structured interviews were undertaken with eighteen married women living in informal or socioeconomically disadvantaged formal neighbourhoods in Aleppo in 2011, a year before the armed conflict caused massive destruction in all these neighbourhoods.

**Results:**

Our findings suggest that the experience of neighbourhood social characteristics is even more critical to women’s sense of well-being than environmental conditions and physical infrastructure. Most prominent was the positive influence of social support on well-being.

**Conclusions:**

The significance of this study lies, first, in its timing, before the widespread destruction of both formal and informal neighbourhoods in Aleppo and, second, and in its indication of the views of women who lived in marginalised communities on what neighbourhood characteristics mattered to them. Further research post-conflict needs to explore how decisions on urban rebuilding are made and their likely influence on health and well-being.

## Introduction

Studying specific neighbourhood effects of living in informal settlements on health and well-being in low- and middle-income countries has received little attention within the wider literature on urban poverty and health (Lilford et al. 2017). Overall, the limited literature on neighbourhood and health has struggled to conceptualise the mechanisms by which neighbourhoods influence health. In a seminal paper, Macintyre et al. (2002) distinguish three types of explanation for geographical inequalities in health: compositional, contextual, and collective. Compositional explanations highlight the characteristics of individuals or households concentrated in particular neighbourhoods. Contextual explanations relate to the opportunities and constraints posed by the neighbourhood’s physical and social environment, encompassing access to clean air and water or housing quality, or availability of health services. Collective explanations relate to cultural and historical characteristics of neighbourhoods: the extent of community support networks, social cohesion, ethnic mix, and social participation (Macintyre et al. 2002). Our paper rests on the World Health Organisation definition of health defined in Alma-Ata Declaration (1978) ‘as a state of complete physical, mental and social well-being, and not merely the absence of disease and infirmity’ (World Health Organisation 1978).

This paper focuses on what Macintyre et al. define as the contextual and collective in exploring the topic of neighbourhood and health in Aleppo on the eve of a national catastrophe, as seen through the eyes of women who are residents of these neighbourhoods. The impact of the seven-year armed conflict on population health has been overwhelming, not least from violent death, injuries and psychological trauma, but also due to widespread destruction of homes, economic hardship, targeting of healthcare facilities, and crucially the damage caused to the social fabric of what was an ethnically and religiously mixed society (Fouad et al. 2017; Taleb et al. 2015).

In Aleppo, 29 informal settlements (out of a total of 114 neighbourhoods classified by the municipality) were home to around one million people in 2004 (i.e. about 40% of Aleppo’s estimated total population of 2.5 million) (Hammal et al. 2005). Informal settlements in Aleppo were defined as meeting one or more of the following criteria (The Aleppo Informal Settlements Task Force 2009, p. 8):

- The ownership of the land is disputed and/or is not legally registered.
- Lack of compliance with the master-plan land use zoning regulations and planning standards.
- The construction of dwellings does not comply with building standards and regulations.

There are a number of reasons for the proliferation of informal neighbourhoods in the decade up to 2011. These included rapid population growth, substantial internal rural–urban migration fuelled by long droughts (2006–2010), economic and social policies that persistently neglected rural areas (Fernandes 2008; Lavinal 2008), and increasing socioeconomic inequalities (Ahmad and Sudermann 2012; Goulden 2011; Hinnebusch 2012b).

As in other Syrian cities, the distinction between the physical environment of ‘formal’ and ‘informal’ neighbourhoods in Aleppo was not clear-cut (Clerc 2014). Many informal areas were served by some degree of public infrastructure (Goulden 2011; Hammal et al. 2005; Lavinal 2008). The continuity between the lowest-income neighbourhoods (formal and informal) is suggested by the fact that only 10% of Syria’s housing stock was rated by UN-HABITAT as slum housing, a low figure for the region (Goulden 2011; Hinnebusch 2012a), and that urban poverty was not confined to informal neighbourhoods (El Laithy and Abu-Ismail 2005). However, informal neighbourhoods were characterised by unsafe buildings, substandard infrastructure, and meagre service provision (e.g. poor refuse collection and sanitation), which in turn have negative public health impacts (Goulden 2011; Hammal et al. 2005).

This paper draws on findings from qualitative interviews with married women living in low-income neighbourhoods (formal and informal) in 2011 before the armed conflict caused devastating destruction. It has two main aims: to explore low-income women’s understandings of the influence of their own neighbourhood on their sense of health and well-being and to offer insights for subsequent policy post-conflict. We believe it is timely for several reasons. Firstly, this is the first in Syria to focus on women living in formal and informal neighbourhoods (Coker 2008; Habib et al. 2009; Hammal et al. 2005; Makhoul et al. 2003). Secondly, exploring the perspectives of marginalised women in impoverished neighbourhoods at the start of the conflict was itself highly subversive, especially in an authoritarian political system in which people had little voice. This account therefore offers a rare insight into women’s perceptions of their lives in these neighbourhoods on the eve of a collapse that also renders these insights prematurely historical. Thirdly, we would argue that reporting these such perspectives is itself an ethical obligation, given the extent of destruction and human loss engulfing these most low-income neighbourhoods in Aleppo, many of which are the very ones researched in this study.

## Methods

The paper reports findings from a purposive sample of eighteen low-income women living in informal and low-income formal neighbourhoods (ten lived in informal neighbourhoods). The qualitative fieldwork informing this study was part of the first author’s doctoral thesis.

Married women were recruited into the study to ensure a diverse mix of neighbourhoods and individual characteristics. Individual diversity was sought in women’s educational attainment; ethnicity and religion; employment status and origin from a rural or an urban area. Neighbourhood diversity was sought to ensure a mix of low-income formal neighbourhoods and informal settlements based on the ethnic and religious composition of the population and the quality of infrastructure. Moreover, participants from the informal neighbourhoods were recruited to represent the three informal regions in northern, eastern and southern Aleppo, see (Hammal et al. 2005) for more details on similarities and differences between informal neighbourhoods in these three zones.

There were three reasons for focusing on married women. First, married women were hypothesised to spend a large part of their daily lives in their neighbourhoods, because the Aleppo Household Survey had indicated that 89% of married women were housewives (i.e. not in formal employment) (Maziak et al. 2005). As a result, married women were assumed to have particular insights into the possible impacts of neighbourhood characteristics on health. Second, it was an opportunity to explore contextual neighbourhood factors that could be associated with women’s self-rated health (SRH). A study on self-rated health and its determinants in Aleppo showed married women were more likely to report poor SRH than the unmarried and more than twice as likely as married men to report poor SRH (Asfar et al. 2007). Third, in practical terms, it was much easier for a female doctoral researcher (first author) to access women in a largely conservative social setting.

The qualitative study took place between April and June 2011, beginning a month after the uprising started. At the time of the study, there were no protests in Aleppo. However, the volatility of the political situation provoked wariness towards researchers linked to western universities, and made conducting research, especially in low-income neighbourhoods, a real challenge. Hence, modifications to the original qualitative data collection plan proved necessary. There was no alternative other than to use the networks of trusted colleagues and friends in the recruitment of study participants and to steer away from undertaking interviews in certain neighbourhoods that were considered particularly unsafe. Ultimately, the qualitative fieldwork had to be terminated earlier than the original plan in order to ensure the safety of the participants and the principle researcher.

Interviews were conducted by the first author (a native Syrian) in colloquial Arabic. These lasted between 45 and 60 min. The following questions were asked to explore how living in low-income (and informal) neighbourhoods influenced women’s health and well-being:

1. How does living in the neighbourhood influence your health?
2. What are the things that most disturb you in this neighbourhood?
3. What do you like most about living in your neighbourhood?

In addition to the questions above, follow-up questions were used as necessary to explore a sense of belonging to the neighbourhood and positive and negative experiences of the locality. All interviews were translated from Arabic into English by the first author. Back translation was undertaken by an independent bilingual translator. Extracts of the translated texts were discussed with the second author of this paper to check inferences and meanings, to enhance overall validity.

Thematic analysis was used (Braun and Clarke 2006). The interview guide for women respondents was used as a basis to develop the coding index. No qualitative software was used in the analysis. The first author undertook all steps in the data analysis and interpretation of the findings. All co-authors provided feedback at various stages of the analysis, from coding to data interpretation and reporting. However, the role of the second author (FMF) was especially important in the interpretation of major themes and for informing a contextual understanding of the political and social history of each selected neighbourhood, given that FMF was a native of the city and had worked with socioeconomically disadvantaged communities for many years as a doctor, director of the city health department, and researcher. Ethical approval was granted by Newcastle University Ethics Committee and the Syrian Center for Tobacco Studies. Verbal consent was sought from the respondents prior to the interviews. Pseudonyms were used throughout.

## Results

The majority of the respondents lived in their neighbourhood for 10 years or more: 6 out of 8 in the formal neighbourhoods and 7 out of 10 in informal neighbourhoods (overall range was 3–37 years). Most women, especially those living in informal neighbourhoods, reported low educational attainment (almost all below ninth grade in school) and four reported being illiterate. Eight out of ten of respondents in informal neighbourhoods originated from rural areas, compared to three out of eight who lived in the formal neighbourhoods.

The findings from the interviews offer an insight into women’s perceptions of the impact of their own neighbourhood on their health. It is worth noting that respondents struggled to answer the question on the direct relationship between neighbourhoods and health. They would initially answer ‘none’ or ‘cannot think of any’ and one expressed surprise that neighbourhoods could influence health. More effective in eliciting responses was the question on the things that most disturb you in this neighbourhood. Findings are grouped under three headings: the physical characteristics of the neighbourhood; its social character; and the impact of urban planning policy, including eviction and demolition policy.

### The physical characteristics of neighbourhoods influencing women’s health and well-being

#### Poor quality housing

Many women described the stress caused by dampness and cold in the inadequate structures of their homes. Um Hishām (the mother of Hishām: in Arab society women and men take on the names of their first male child), lived in an informal neighbourhood in the southern part of Aleppo with a majority Arab Muslim population. She described the financial implications of living in a cold and poorly located home, especially being the breadwinner supporting a large family:

There is a lot of dampness in my house. I hate my life because of it. The electricity bills are high. I still do not know how I am going to pay them. When it rains heavily, the sewer floods and the house fills with filth.

Her views were echoed by other respondents from different ethnicities and religions living in low-income formal and informal neighbourhoods. Amani, a 49-year-old woman, who lived in a low-income formal neighbourhood (largely Christian), spoke of flooding and humidity:

My house is a ground floor flat and it has no outside space. I need to stand next to the front door to see people…. Also the flat is very humid…. My flat gets flooded when it rains.

Similarly, Nivīn, a 42 year-old Kurdish housewife who had moved with her large family from a rural area to rent in a very low-income informal neighbourhood in north Aleppo, deplored the problems of her home:

There is considerable dampness in the house. Nothing works. There is no ventilation. The place is very small and we share the kitchen and toilet.

#### Pollution: health risk, sign of social and physical neglect, or normality?

When residents spoke of pollution problems (a minority), they mentioned the unregulated disposal and accumulation of waste in their neighbourhood, but nothing else (air or water quality for example). Even then, only one woman spoke of waste as a potential health hazard, and she was unusual in having had 2 years of college education. Hāla lived in an informal neighbourhood in northern Aleppo, with a majority of Palestinian refugees, and linked waste to leishmaniasis (also known as Aleppo boil):

There is a lot of garbage in the street. Also Aleppo boil is very prevalent in the area.

More common was to moralise the issue of waste disposal as typifying the casual or neglectful behaviour of others, including other groups, rather than seeing it as an infrastructure or organisational problem. Narimān, who lived in a formal neighbourhood with a Christian majority, offers an illustration, in her complaints about the conduct of those around her:

There is no cleanliness here. People throw their waste onto the street from the balconies. This is all laziness. They do not like to bother themselves by going downstairs.

By contrast, Khadījah, a 29-year-old Arab woman living in a majority Kurdish informal neighbourhood in north Aleppo highlighted how those from rural backgrounds had become habituated to certain kinds of pollution:

We the inhabitants of villages are used to noise and dust.

### The social characteristics of neighbourhoods influencing women’s health and well-being

#### The importance of the religious and ethnic composition of a neighbourhood

The importance attached to living in a neighbourhood with those from the same religious background was most apparent among Christians. Some spoke not only of their preference for living in neighbourhoods where other Christians lived but also the desirability of having their church nearby. In Narimān’s words:

I feel comfortable in the area. I like it, when I wake up in the morning and see the church [she lived opposite to the church] in front of me. When I wake up and see the cross I relax.

Not all women felt this need and some preferred more mixed neighbourhoods. Um Samīr, for example, identified herself as a Muslim. For her the large Christian community in the low-income formal neighbourhood where she lived was an important consideration when she decided to move a decade ago when she was widowed. Um Samīr linked the presence of a large Christian minority with her and her daughters feeling safe. She mentioned how she was prepared to pay a higher rent to live in this neighbourhood rather than move to a much cheaper but traditional and conservative neighbourhood where women, especially those who did not cover up, might be stalked. Um Samīr pointed to the liberal code of dress of her daughters and how important for her well-being and that of her daughters that they could maintain their liberal values without fear of being harassed or stalked. She referred to the protective nature of the Christian males in her street who knew them as a Muslim family:

Men are very protective to girls of their neighbourhood and they do not allow any stranger to look at them or stalk them. They have always been very good to us.

The importance of social networks in a neighbourhood came to the fore when women from informal neighbourhoods composed of large ethnic minority communities (mainly Kurdish or Palestinian) described the presence of the extended family nearby, and in many cases referred to strong social support from neighbours (see Table 1).

**Table 1:** Examples of perceived social support in informal neighbourhoods in Aleppo, Syria (2011)

“She comes every other day. She may not visit her parents for a week but not me. Two days ago she came and found me really tired, and she cleaned the house for me. She washed all the dishes. She even tidied up the whole house for me”. (Khadija, lived in a largely Kurdish informal neighbourhood) “I have three neighbours of mine whom I have a very strong relationship with. If anything happens they ask about me always”. (Hind, lived in a mainly Palestinian informal neighbourhood)	

#### Manṭiqah sha‘bīyah’ (working-class or ‘populous’ neighbourhoods)

No local idiom conveyed more than the Arabic term ‘*manṭiqah sha‘bīyah*’. Used of low-income neighbourhoods, it has no simple translation and ambiguous connotations that could be positive or negative. It appeared widely in women’s narratives when describing the nature of social relationships in the neighbourhood and its influence on their well-being. ‘*Manṭiqah sha‘bīyah*’ conveyed a sense of what is ‘common’ or ‘popular’, but variously seen as desirable or undesirable. As a descriptive term applied to a neighbourhood, it might characterise a popular area appealing for a vibrant sense of community, a sociable place, or alternatively one that was stigmatised as associated with very low-income populations (for example, those from traditional conservative communities (mostly Muslim) or certain rural areas).

The majority of respondents living in informal neighbourhoods used *manṭiqah sha‘bīyah’* to speak favourably of neighbourhood attributes with positive implications for their well-being. Some identified such neighbourhoods as providing a high degree of social support and a feeling of safety. Hind, for example, lived in an informal neighbourhood in northern Aleppo with a majority of Palestinian refugees and pointed to the social support she saw around her:

I love manaṭiq sha‘bīyah’. There is so much good will in these areas. People look out for each other in these areas, unlike in the city. I used to live in the city. If anything happened to you, no one would pay you a call. No one will know what has happened to you. The best thing I like in my area is that people are there for each other.

But there were others—a minority—for whom the term was used less positively. Najwá, for example, a Muslim woman with meagre means, lived in an extremely conservative informal neighbourhood that attracted low-income manual workers originating from different rural or urban areas in Syria. For her ‘*manṭiqah sha‘bīyah’* implied incivility more than sociability, and she distanced herself socially from those around her:

[neighbourhood name] is an extremely ‘working-class’ neighbourhood, and there is delinquency and incivility. Most people who live here are unpleasant and the children misbehave and are violent.

In Table 2 we present quotes from two women, both widows of different ethnicity and religion but similar socioeconomic status (they worked informally as cleaners). It was striking to note the contrasting connotations of *manṭiqah sha‘bīyah* in these women’s descriptions of their neighbourhoods.

**Table 2:** Two contrasting views of Manṭiqah sha‘bīyah’ (working-class or ‘populous’ neighbourhoods), Aleppo, Syria (2011)

“…The neighbourhood is a manṭiqah sha‘bīyah (Working-class or ‘populous’ neighbourhood) in the centre of Aleppo. It is quite safe. The most important thing is its safety”. (Um Samīr, an Arab Muslim widow, 45 years old) ’This is a manṭiqah sha‘bīyah. (Working-class or ‘populous’ neighbourhood). I would not live in it if I had the economic means to move elsewhere”. (Kristīna, an Armenian Christian widow, 52 years old)	

## Discussion

This is the first qualitative study from Syria to explore the views of low-income married women living in informal and other socioeconomically disadvantaged neighbourhoods on the characteristics of those neighbourhoods that concerned them and had the potential to impact on their health and well-being. In a Syrian context, where research has been limited, Aleppo has had more attention than other cities. This paper complements and expands on findings from previous published qualitative studies on informal settlements in Aleppo (Ahmad 2014; Ahmad et al. 2009, 2013; Ahmad and Sudermann 2012; Hammal et al. 2005; Maziak et al. 2005).

The findings suggest that women’s experiences of their neighbourhood’s characteristics are central to their sense of well-being. With isolated exceptions, they did not make a link between the physical environment they lived in and their own health. Irrespective of the formality of their neighbourhood, women pointed to a range of neighbourhood physical and social characteristics that either enhanced or reduced their stress and well-being.

Our findings are similar to those from qualitative studies in urban settings of developed countries (Bolam et al. 2004; Castro and Lindbladh 2004; Davidson et al. 2008; Popay et al. 2003; Wallis et al. 2010) in that poor housing quality and inappropriate waste disposal featured prominently in women’s accounts of the physical features of neighbourhoods that were perceived to influence their well-being. However, a notable difference was that women in our study hardly referred to physical health problems that they associated with the conditions in which they lived. Whether this reflects the limited educational backgrounds of these women we could not say. However, it is in line with findings from earlier studies in this setting which showed illiteracy and low educational attainment to be highly prevalent among low-income women, especially those living in informal settlements (Ahmad and Sudermann 2012; Hammal et al. 2005; Maziak 2009; Maziak et al. 2005). The finding may also reflect the normalisation of some of the effects of the physical environment on health. This confirms observations made in earlier studies from Aleppo involving two of the current authors (Ahmad and Sudermann 2012; Hammal et al. 2005). It is also worth noting that despite the perceived poor quality of housing in informal neighbourhoods a very small proportion of informal housing at the time of the study fitted the UN-HABITAT definition of slum housing (UN-HABITAT. 2003), an important technical issue discussed elsewhere (El Laithy and Abu-Ismail 2005; Goulden 2011).

This emphasis on well-being rather than health, and on social networks rather than the physical infrastructure of their environment, may well reflect these women’s sense of where their own limited agency lay. Making and sustaining their social networks was within their sphere of influence; changing their immediate physical environment was not. This finding seems to support recent work during the present conflict. For instance, one recent study explored the impact of the armed conflict on social capital, reporting a decline compared to pre-conflict (Syrian Center for Policy Research 2017). This study attributed this deterioration to increased feelings of insecurity, to increasing poverty, forced displacement—and a deterioration in living conditions. Acknowledging the transformed role of women in bearing the economic burden of a family, the report equally highlighted women’s increased marginalisation, where ‘political oppression, extremism, widespread violence, and exploitation’ compromised women’s possibility of social participation (Syrian Center for Policy Research 2017, p. 6). We add our recommendations to existing ones to promote the use of social capital building approaches, such as community-driven development, job creation, and education as peace building blocks in post-conflict settings (Idres 2016; Sibai et al. 2017). This is a topic that will require considerable further research, as post-conflict rebuilding becomes possible in urban Syria.

The qualitative fieldwork on which this study was based took place during the early months of the Syrian crisis, before its full impact had reached Aleppo. As a result, the key limitation was the curtailed scope of the study, reflecting the deteriorating political situation at the time. Few studies can be undertaken in such difficult circumstances amidst rising tension. However, the timing of this study offsets its small scale. For our findings also provide a rare glimpse into factors which contribute to or jeopardise the well-being of women from the most marginalised parts of Aleppo, on the eve of a conflict that would engulf their city and tear their lives apart.

Thus our study reaffirms the importance of including the voices of women in marginalised communities in informal and low-income urban neighbourhoods in post-conflict urban planning and policy. This is crucial with future urban renewal policy in Syria shifting from the upgrading of informal settlements to outright demolition and reconstruction, a shift that predates the conflict but has become even more politicised post-conflict (Clerc 2014; Wakely 2010). For example, Law 10 (enacted in April 2018) and its predecessor Decree (66), a Damascus-only urban reconstruction decree, draw regulatory master plans and mega projects to ‘formalise’ informal neighbourhoods (Hanna 2018). Both laws advance the government agenda for urban reconstruction by inviting national and international investors at the expense of evacuating residents from their homes, offering inadequate compensations in the form of shares and ignoring property rights (Hanna 2018). In a case of an informal settlement in the capital Damascus, the municipality ordered residents to evacuate their homes, promising them compensation, substitute housing and shares in what will become modern tower blocks that will replace their homes and businesses (Al-Sabouni 2016). We recommend that empirical research is undertaken to explore claims of the *weaponisation* of urban planning policies in the era post-conflict (Clerc 2014; Hanna 2018), and the impact on health and well-being of low-income marginalised communities in a way similar to the recent investigation by the *Lancet*-*American University of Beirut Commission on Syria: Health in Conflict* into the *weaponisation* of healthcare during the armed conflict (Fouad et al. 2017).

In underscoring the importance of neighbourhoods in the health and well-being development agenda, and the need to consider voices of marginalised groups including women in urban planning, we argue that the road to community health and well-being is the same as that for social peace: both must start with ‘education, equality and security’ (Maziak 2006, p. 815).
